# Quantifying temporal change in biodiversity: challenges and opportunities

**DOI:** 10.1098/rspb.2012.1931

**Published:** 2013-01-07

**Authors:** Maria Dornelas, Anne E. Magurran, Stephen T. Buckland, Anne Chao, Robin L. Chazdon, Robert K. Colwell, Tom Curtis, Kevin J. Gaston, Nicholas J. Gotelli, Matthew A. Kosnik, Brian McGill, Jenny L. McCune, Hélène Morlon, Peter J. Mumby, Lise Øvreås, Angelika Studeny, Mark Vellend

**Affiliations:** 1 Scottish Oceans Institute and Centre for Biological Diversity, School of Biology, University of St Andrews, East Sands, KY16 8LB, UK; 2 Departamento de Biologia, CESAM, Universidade de Aveiro, Campus de Santiago, Aveiro 3810, Portugal; 3 CREEM, University of St Andrews, The Observatory, Buchanan Gardens, St Andrews, Fife KY16 9LZ, UK; 4 Institute of Statistics, National Tsing Hua University, Hsin-Chu, Taiwan 30043; 5 Department of Ecology and Evolutionary Biology, University of Connecticut, Storrs, CT 06269, USA; 6 Department of Civil Engineering and Geosciences, Newcastle University, Newcastle upon Tyne NE1 7RU, UK; 7 Environment and Sustainability Institute, University of Exeter, Treliever Road, Penryn, Cornwall TR10 9EZ, UK; 8 Department of Biology, University of Vermont, Burlington, VT 05405, USA; 9 Department of Biological Sciences, Macquarie University, Sydney, New South Wales 2109, Australia; 10 School of Biology and Ecology and Sustainability Solutions Initiative, University of Maine, Orono, ME 04469, USA; 11 Department of Botany and Biodiversity Research Centre, University of British Columbia, Vancouver, CanadaBC V6T 1Z4; 12 Center for Applied Mathematics, Ecole Polytechnique, UMR 7641 CNRS, 91128 Palaiseau, France; 13 Marine Spatial Ecology Laboratory, School of Biological Sciences, University of Queensland, St Lucia, Queensland 4072, Australia; 14 Centre for Geobiology, University of Bergen, 5020 Bergen, Norway; 15 Centre de recherche INRIA Grenoble - Rhone-Alpes, Inovallée, 655 avenue de l'Europe, Montbonnot, 38 334 Saint Ismier CedexFrance; 16 Département de biologie, Université de Sherbrooke, Sherbooke, Québec, CanadaJ1K 2R1

**Keywords:** biological diversity, time, legacy data, traits, global change, conservation

## Abstract

Growing concern about biodiversity loss underscores the need to quantify and understand temporal change. Here, we review the opportunities presented by biodiversity time series, and address three related issues: (i) recognizing the characteristics of temporal data; (ii) selecting appropriate statistical procedures for analysing temporal data; and (iii) inferring and forecasting biodiversity change. With regard to the first issue, we draw attention to defining characteristics of biodiversity time series—lack of physical boundaries, uni-dimensionality, autocorrelation and directionality—that inform the choice of analytic methods. Second, we explore methods of quantifying change in biodiversity at different timescales, noting that autocorrelation can be viewed as a feature that sheds light on the underlying structure of temporal change. Finally, we address the transition from inferring to forecasting biodiversity change, highlighting potential pitfalls associated with phase-shifts and novel conditions.

## 1. Introduction

A key scientific challenge is to quantify and forecast temporal change in biodiversity attributable to both natural and anthropogenic causes [1,2]. Forecasting biodiversity change is essential for developing successful policies to mitigate biodiversity loss [3] and for addressing basic ecological issues, such as the relationship between diversity and ecosystem function [4], the linkage between diversity and stability [5] and the detection of ecological tipping points [6] in relation to the existence of alternative stable states [7]. Because most biodiversity studies are observational rather than experimental—particularly at large scales, we argue that temporal relationships between biodiversity, ecosystem services and hypothesized driver variables are among the strongest possible evidence for causal links. Moreover, temporal studies of biodiversity are essential for forecasting future change in community structure and ecosystem function.

We begin by discussing key characteristics of biodiversity time series, presenting details on the advantages and limitations of different data sources in the electronic supplementary material. Second, we address the quantitative analysis of biodiversity time series, identifying four main factors affecting observed biodiversity temporal change: measurement error, process error, systemic change and historical influence. We discuss methods used to estimate, quantify or (when appropriate) minimize these sources of change. Third, we highlight approaches and potential pitfalls in forecasting biodiversity change, on the basis of inferences drawn from past trends. We are restricted to time series of one (any) quantitative metric of biodiversity. We are purposely agnostic about which metric, and illustrate that the same analysis tools can be used for different metrics. We highlight that anecdotal evidence and historical records can provide important information, which need only be translated into a quantitative assessment for these tools to be useful for this sort of data.

## 2. Characteristics of temporal biodiversity data

**Figure 1.**
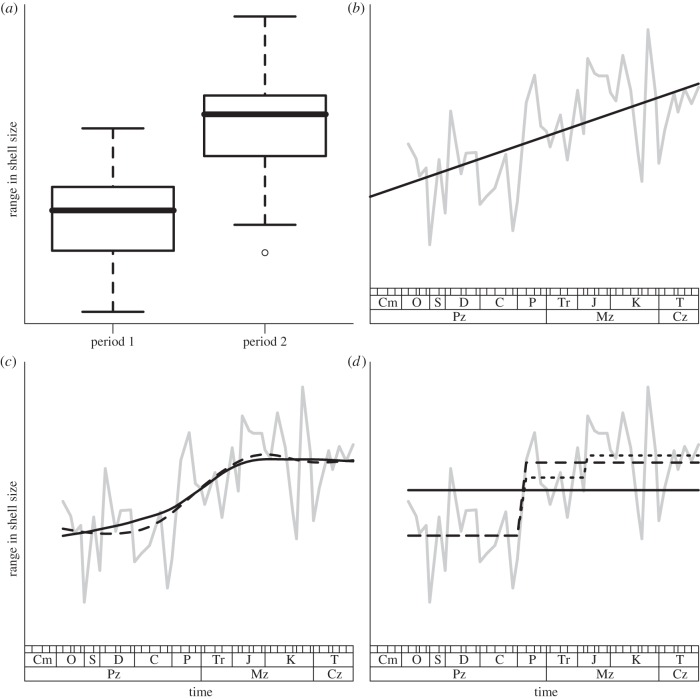
Four ways of analysing trends in biodiversity. The data are range in gastropod fossil shell size (a metric of trait diversity) through the Phanerozoic from Kosnik *et al*. [8]. Similar figures analysing taxonomic and genetic diversity are included in the electronic supplementary material, figures S4 and S5 to illustrate how similar analysis tools can be used for different components of biodiversity. Grey lines show the observed data. All analysis done in R v. 2.12.2 (90); the code is included as electronic supplementary material. (*a*) *t*-test comparing shell size diversity in two time intervals (plotted as a box plot) with observed mean shell size range significantly different at *p* < 0.001. (*b*) Global trend analysis; a linear trend is fit using both ordinary least squares (OLS; which ignores the non-independence of errors close in time, black solid line), and generalized least squares (GLS) using a model with AR1 temporal autocorrelation of errors (dashed line). The two lines estimated by the two methods are identical; hence, only the solid line is visible. The main difference in the two models is for the *p* value with *p* = 0.007 for the OLS and the more conservative and correct value from the GLS of *p* = 0.033. (*c*) Local trend analysis; local regression using LOESS smoothing (black solid line) and a GAM spline model (dashed line) of richness versus time are plotted. The results are similar with both methods suggesting that the change in trait diversity over time is nonlinear. (*d*) Threshold regression [9] to formally identify both the number and location of breakpoints. The plot shows the null model of no threshold (black solid line), the preferred model of one threshold break (dashed line) and the second best model of two thresholds (pointed line). The preferred model shows a *Δ*BIC of >8 versus the null model showing very little evidence to select the null model. An *F*-test also shows the null model rejected at *p* < 0.001. Similar figures are included in the electronic supplementary material, using examples of genetic and taxonomic diversity on the *y*-axis.

A biodiversity time series documents the abundances (or at least presence–absence) of multiple genes, traits or taxa at multiple points in time. Taxa—species, in particular—are the most common units of diversity, but most of the methods we discuss are also applicable to other units of diversity (see figure 1 and electronic supplementary material, figures S4 and S5 for an illustration of this point). These data are typically used to estimate one or more biodiversity metrics at each time point. Common diversity metrics include species richness (the total number of species), evenness (the relative dominance of taxa), species diversity (indexes that combine both richness and evenness), functional diversity (the range of traits present in the community, which are often responsible for ecosystem function), phylogenetic diversity (the evolutionary breadth of the community) or compositional analysis. The merits of different biodiversity metrics have been thoroughly discussed elsewhere [10].

Collecting (or assembling) temporal data involves distinct challenges from investigations made using spatial data. In space, the grain (the units of observation), extent (the universe encompassed by the data) and coverage (the proportion of the extent that is observed) [11] can always be adjusted, assuming that sufficient resources are available. However, researchers cannot travel in time, and so must be opportunistic and creative in identifying temporal data sources.

Four sources of data can be used for temporal inference: temporally replicated sampling, chronosequences (in which space is used as a proxy for time), legacy or historical records and palaeobiological assemblages (see the electronic supplementary material). Integrating data from different sources can provide insights not possible to get from any one source and may overcome some of the weaknesses of each type of data. For example, a comparison of temporally replicated sample data with chronosequences can directly test the validity of the space-for-time substitution [12]. Also, combinations of multiple time series, including palaeobiological, historical and contemporary data, can extend time series or provide more frequent sampling [13].

Temporal data differ from spatial data in at least three crucial characteristics. First, temporal data are directional, which creates an asymmetry in the relationship among data points: the past can influence the future, but not the reverse. This critical property of temporal data can be used to strengthen inference about causality [14] because effects cannot precede causes. This asymmetry in the cause–effect relationships can be used to predict change. Additionally, the statistical estimation of time lags can shed light on cause-and-effect relationships in temporal data.

Second, time is uni-dimensional, whereas space has three dimensions. In this respect, strictly temporal patterns are simpler to analyse than spatial patterns. In fact, spatial patterns are often collapsed into fewer dimensions, such as transects along latitudinal, topographic and habitat gradients [15]. However, time and space are frequently confounded, as in historical fisheries records that cover time periods when the fleet was focusing on different areas, or palaeo records that cover different spatial locations as well as periods of time. Every time series is embedded in a spatial context, just as every spatial dataset is embedded in a temporal context. Hence, it is important to either assess change in a spatio-temporal context or to consider the contribution of spatial variation in the time series to measurement and process error (see §3).

Third, temporal domains are often unbounded because, in principle, the beginning and end of a time series is arbitrary. However, there are several potential ‘natural’ boundaries to time series, including colonization of new space, adaptive radiations, the annihilation of a community (e.g. continental glaciation or mass extinction), sharp transitions into alternative states and the present day. In spatial data, boundaries can directly or indirectly generate strong signals. For example, even if species are randomly distributed within a spatial domain, geometric constraints in range distribution lead to a non-uniform accumulation of species at the domain centre (the mid-domain effect [16]). These patterns would not be expected to occur on unbounded temporal series. For bounded time series, directionality means that the effect of a starting boundary is different from that of an ending boundary. The starting point is an important part of the successional pattern that follows [17]. Moreover, when studying temporal change relative to an arbitrary starting point, sensitivity of the conclusions to the chosen baseline needs to be considered, and potential effects of a shifting baseline should be recognized [18].

Temporal and spatial datasets also share some qualities. The concept of grain [11,19] is equally applicable to spatial and temporal data. For temporal data, grain size is the degree of time averaging within each data point, which is akin to spatial averaging where biodiversity is quantified within an area, rather than at a single point in space. In practice, almost all data include some component of both temporal and spatial averaging because spatial data are seldom simultaneously collected in a single ‘snapshot’, and temporal data are rarely collected at exactly the same spatial location. Grain size can be standardized across multiple time series by temporally averaging higher resolution series, or it can be statistically controlled in the analysis [20]. Census interval (the time period between two discrete samples) also affects temporal resolution. Increased census interval tends to be associated with increased temporal turnover [21,22].

## 3. Analysing temporal change

Regardless of the methods used to gather data (see the electronic supplementary material), observed temporal change in biodiversity can be attributed to four main factors: measurement error, process error, historical influence and systemic change. Measurement error includes sources of apparent change that reflect bias or imprecision in measurement (including detection error), and can reduce our ability to identify patterns of interest. Process error refers to mechanisms that are not included in the model, and is different from measurement error. Historical influence is reflected in the patterns of temporal autocorrelation of the biodiversity time series. Typically, we are interested in understanding the effects of particular drivers of interest on systemic change. Systemic change reflects a non-stationary system in which there are long-term changes in ecological drivers, both anthropogenic (ongoing climate change and increases in nutrient deposition) and natural (shorter-term successional change and long-term changes in speciation and extinction rates). Temporal change due to other drivers may occur as a result of process error, and this partitioning depends on the questions being addressed. Explicitly recognizing sources of error allows the investigator to statistically control for these when testing for systemic change in a biodiversity time series (see the electronic supplementary material, figure S1 for an example in which seasonal variation is removed to focus on longer-term trends).

Each temporally based observation of biodiversity arises from the combined effects of deterministic and stochastic drivers of change. Ultimately, the processes involved in systemic change depend on the component of biodiversity being studied and the spatial and temporal extent of the data. At the most general level, the main processes behind change within and among species are mutation, drift, selection, dispersal, speciation and extinction [23]. In order to draw inferences about how different predictor variables affect these processes and to forecast biodiversity change, measurement and process error must be minimized or estimated, and historical effects must be understood [24].

### (a) Dealing with measurement error

Measurement error is often the elephant in the room: everyone who has collected empirical data is aware of its existence, but we are sometimes reluctant to discuss its presence for fear it undermines the credibility of results. However, identifying and quantifying measurement error minimizes its effects on drawing inference. Moreover, reporting relevant sources of measurement error stimulates the development of methods to minimize or control for error, and allows future data users to make informed decisions about how to learn from data.

Measurement error varies too much among biodiversity components and potential drivers of biodiversity change for a comprehensive review, here, of its sources and the tools available to minimize it. Some examples are presented to illustrate the variety of sources of measurement error. Instruments that measure environmental data have associated measurement error, which may change along a time series as different equipment can have different precision and accuracy. For taxonomic diversity, sources of measurement error include misidentification of specimens, changes in nomenclature, failure to recognize cryptic taxa and variation in detection probabilities among taxa [25,26]. For trait diversity, measurement error arises from error in the physical measurement of traits or inconsistency in trait measurements [27]. The latter is particularly affected by ontogenetic and phenotypic plasticity, which may create false signals if appropriate standardization is not used (e.g. a temporal trend in leaf morphology due only to plant age). For genetic diversity, sources of error are associated with the processes of selection of the genes of interest, amplifying and sequencing genes, and (especially for microbes) determining the boundaries of operational taxonomic units. In the case of phylogenetic diversity, error associated with the process of building (including topology and branch lengths) and dating molecular phylogenies must also be considered. Finally, some sources of error are common to all biodiversity components, such as misinterpretation of records, mistakes in transposing information and sampling error.

The most prevalent source of measurement error in biodiversity data is that most biodiversity metrics are sensitive to sampling intensity [28]. Observed species richness, for instance, is an underestimate biased against rare species, which typically comprise the greatest fraction of species. Criteria of rarity in a spatial context include the abundance at any one location, spatial occupancy and habitat specialization [29]. Biodiversity time series have an additional criterion, the probability of occurrence over time (i.e. transient versus resident species [30]). Similar reasoning applies to traits and alleles, although abundance distributions of these are less well understood. In theory, sampling at a site could continue until an asymptote is reached, but in practice, this is seldom possible. Hence, although sampling intensity should be as high as feasible, meaningful comparisons can be made only if sampling effort is standardized either while collecting data or statistically.

The two main strategies to standardize data statistically, for any form of comparison including temporal comparisons, are subsampling and extrapolation [31]. Rarefaction to a common sampling effort adjusts for differences in sampling intensity, and has long been used with palaeontological time series [32]. The chief disadvantage of rarefaction is the loss of information involved in equalizing sample size to the smallest sample in the time series. An alternative is to adjust sampling effort according to the diversity of the community being sampled [33,34]. The Good Turing concept [35,36] suggests that observed rare entities carry most information about the undetected diversity in a sample. Hence, rather than using uniform sample sizes, this method proposes adjusting sampling effort to achieve proportionally similar samples in order to decrease bias in richness estimates. This implies higher sampling effort when there are many rare species [37,38]. Another approach is extrapolating to estimate the asymptote of the sampling accumulation curve [39]. This approach has been designed for species richness but can be applied to other components of biodiversity. Several methods are available for doing this, including asymptotic curve-fitting [40], parametric estimators based on abundance distributions [41,42] and non-parametric estimators [43,44].

Sampling methods have inherent biases that cause some taxa, traits or genes to be detected more readily than others. Estimating detectability can improve the accuracy of abundance estimates [45], although all sampling methods have biases [28]. The simultaneous use of multiple sampling methods can reduce some of these biases [46]. In temporal studies, sampling methods are not always controlled by the scientist throughout the time series, particularly when using historical or large-scale data. In this case, it is necessary to control for the effect of sampling method statistically, by standardizing the time series with respect to the sampling bias of each method [47].

### (b) Historical effects: understanding temporal autocorrelation

Autocorrelation can be an important reason to be wary that ‘correlation is not causation’, but time series can be particularly informative in assessing causality because the timing of events makes it possible to deduce the direction in which information is being transferred [48]. Both temporal and spatial data are affected by autocorrelation, with points closer in space or time on average more similar than distant points. As a result, at least in realistic ecological situations, variability typically increases with increasing extent [49]. However, the nature of autocorrelation differs between time and space in three subtle ways. First, a focal point in space can influence and be influenced by nearby points in three dimensions, whereas a focal point in time can be influenced only by points that precede it and can only influence points that follow it chronologically. This does not necessarily mean that spatial autocorrelation is stronger, because effects on a focal point from different directions can be counteracting. Because of the three dimensions, there is also the possibility of anisotropy (different covariances in different directions) in space but not in time. Second, the underlying autocorrelation in time, arising at least in part because some or all organisms survive into the next time period, is generally intrinsically stronger than any type of spatial influence, where the most direct causal factor is dispersal or environmental autocorrelation. Third, from an empirical point of view, cycles are common and important in temporal but rare in spatial autocorrelation patterns.

**Figure 2.**
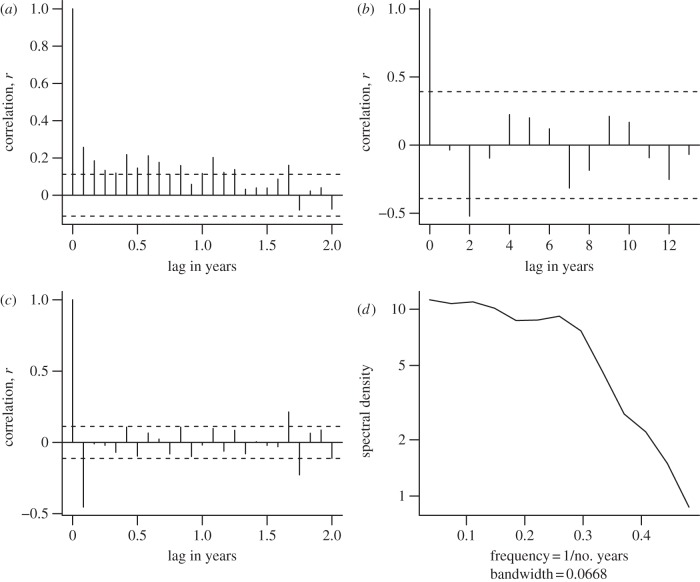
Tools for assessing temporal autocorrelation. These data examine changes in species richness of a small rodent community over 26 years at the long-term research site in Portal, Arizona run by James Brown, Morgan Ernest and others [50,51] at control un-manipulated sites. All analysis done in R v. 2.12.2 [52]; the code is included as electronic supplementary material. The data are monthly or yearly and detrended (via the difference operator) or trend-retained, as described in the titles. (*a*) Autocorrelation function (ACF) analysis on monthly data, with the expected decay of correlation (*y*-axis) with increasing time lags (*x*-axis). (*c*) The same dataset after removing the trend via differencing, highlighting 5-month cycle (these patterns can also be seen in the trend-retained data but less obviously). (*b*) Analysis of yearly data, with a recurring positive signal at approximately 4–5 years (and again at 9–10 years) with matching negative correlations at 2, 7 and 12 years. (*d*) Periodogram on yearly differenced data. The *x*-axis is frequency (the reciprocal of the lag found in ACF plots, i.e. frequency = 1 per lag) and the *y*-axis is a measure of the statistical power found at that frequency. The subtle peak at frequency 0.2–0.3 (=lag of 5–4 years) identifies the same 4–5 year cycle found in the ACF.

In practice, the study of autocorrelation in space and time typically differs in three ways. First, temporal data are typically collected at constant time intervals, allowing easy calculation of lags between points, whereas spatial variables are often recorded at irregular locations distributed continuously in space, requiring the use of techniques such as binning distances to estimate variograms. Second, for historical reasons, variograms (based on variance) are typically used for spatial autocorrelation, while correlograms or autocorrelation function (ACF) plots based on correlation are used for time. Third, the uni-dimensionality of time series, in combination with the prevalence of cyclic changes, means that spectral analysis (see below and figure 2) is often done in time but rarely in space.

There are contrasting perspectives on the implications of autocorrelation for ecological and biodiversity analysis. One perspective is that autocorrelation can lead to spurious conclusions such as inferring a causal relationship between two variables that are correlated only because the observations were non-independent [53]. Thus, autocorrelation must be taken into account when analysing time series to avoid inflated type I error probabilities. This can be dealt with either by removing autocorrelation from the data before the analysis [54], or by using statistical approaches that relax the assumption of independence between observations, such as generalized least squares (GLS), with covariance decaying with distance between points [55]. Another tool specifically designed for this purpose is autoregressive integrated moving average (ARIMA), often used to model and forecast economic time series [56]. ARIMA models can include the following as predictors for a variable at time *t*: various lagged values of the time series, autoregressive terms (i.e. lags of the differenced series) and lagged forecast errors (using a moving average to estimate a local mean instead of the most recent observation). Combinations of these models for different lags typically reduce the influence of autocorrelation on the estimate of the time series global trend.

An alternative perspective is that autocorrelation is not a nuisance, but rather a revealing signal of underlying processes. For example, an analysis of a desert rodent community (figure 2) shows a cycle of autocorrelation approximately every 4–5 years, which is likely related to the influence of El Niño Southern Oscillation on the environment in this region [50]. Patterns of autocorrelation can be quantified by different methods. The simplest way to study temporal autocorrelation is examining the correlation of a time series with itself at different lags (figure 2*a–c*) using ACF analysis [57]. The degree of inertia in the time series can be determined by examining the rate of decay of correlation with time lag. If the time series is long enough and has sufficient resolution, it may be possible to identify temporal cycles by looking for consistent distances in time lags between positive and negative correlations. The time-series spectral density indicates the contribution of different frequencies to the total signal (figure 2*d*). Spectral density can be examined in periodograms, which are typically obtained by using a fast Fourier transform to decompose a time series into sine waves of different frequencies [58]. Important frequencies have a higher density in the periodogram, and its overall shape reflects the type of temporal fluctuations in the system.

Studying autocorrelation as a phenomenon in itself provides crucial insights into biodiversity dynamics and can help increase the accuracy of forecasts of biodiversity change. For example, given that the pattern of autocorrelation in stochastic variation influences population persistence [59], quantifying autocorrelation patterns by examining spectral density of time series may help predict extinction probabilities. More intense, high-frequency variation should increase extinction probability. Moreover, autocorrelation patterns provide indications of relevant external forcing variables. Large-scale climatic variables are often good predictors of temporally autocorrelated patterns in ecology [60], which means that forecasting biodiversity change can take advantage of predicted changes in these variables. In general, incorporating spatial and temporal autocorrelation tends to improve model predictive power [61].

### (c) Quantifying systemic change

Standardizing data to minimize the effects of measurement error and characterizing or removing temporal autocorrelation facilitates quantification of systemic change in biodiversity. However, there is still process error to consider, which can make difficult the task of quantifying systemic change. In practice, disentangling systemic change from process error largely depends on the question being addressed. We distinguish the following approaches to quantifying systemic change: point or interval comparisons, models for temporal data (including long-term and short-term trends) and spatio-temporal models.

#### (i) Comparing points or time intervals

Comparing biodiversity at two points or intervals in time requires an estimate of the precision of the point estimates, typically in the form of a confidence interval. Unless the statistical distribution of the diversity metric is well understood, it is preferable to estimate confidence intervals via a non-parametric bootstrap [62], where sites, species or individuals can be re-sampled, depending on the nature of the data [63]. By plotting the point estimates of diversity with their confidence intervals against time, we can examine temporal changes in the index. Confidence levels must be adjusted when more than two points are compared simultaneously [64]. Inference about the significance of a difference in mean values should consider that significance may be found despite overlapping confidence intervals [65]. Figure 1*a* shows that range in shell size (a metric of trait diversity) was significantly lower in the Ordovician to Carboniferous period than in the Permian to recent period.

#### (ii) Models of temporal trends

An alternative to following the fluctuations of the point estimates is to estimate long or short-term trends in biodiversity. Long-term trends are typically estimated by the slopes of linear regressions of the biodiversity metric over time, whereas nonlinear models can be used to characterize fluctuations and shorter-term trends. Figure 1*b* shows a long-term increase in shell size diversity in a Phanerozoic fossil time series. We show linear trends as fitted by ordinary least squares, that ignores the non-independence of errors close in time, and GLSs using a model with temporally autocorrelated error. Although the lines estimated by the two methods are very similar, the GLS model has a more conservative *p*-value because it models the non-independence of points.

In quantifying systemic change in biodiversity, there are two options to deal with temporal autocorrelation. If seen as nuisance, autocorrelation can be removed *a priori*, for example by analysing ARIMA residuals of the time series or by differencing the data by subtracting successive elements in the time series. Alternatively, the raw data may be analysed, and if the residuals of the model display an autocorrelated pattern, additional predictors may be added to the model to help reduce or remove autocorrelation. Other approaches include modelling residuals as a correlated ARIMA time series or modelling the covariance pattern in the variance-covariance matrix as in the GLS regression. Among many statistical models, generalized additive models (GAMs) [66] are widely used to fit smooth curves or surfaces to data over time for this purpose. GAMs extend generalized linear models and assume additive relationships among the effects of predictors, allowing data to determine the (generally nonlinear) relationship between the response variable and the set of predictors (see electronic supplementary material for more detail and extension into the spatio-temporal case).

A common method for short-term trend models is to use cubic regression splines to construct each smooth function, applying the penalized regression spline technique [67], which controls the degree of smoothness by adding a penalty to the likelihood function. This model usually provides a better fit than parametric linear or quadratic models. Many other smoothing methods are available, including piecewise regression, kernel methods, LOESS (locally weighted polynomial regression), running-mean (or running-median) smoothers, classification and regression tree, and multivariate adaptive regression splines [66,68]. Figure 1*c* shows two local models (LOESS and GAM with splines) fitted to the fossil shell size diversity time series. A comparison with figure 1*b* illustrates the complementary nature of global and local models: despite a long-term increase in diversity of this trait, the rate of change has not been constant.

Non-parametric smooth functions are not only sufficiently flexible to model changes in trends, but also allow us to determine points in a time series at which the rate of change increases or decreases (i.e. the second derivative of the curve). Alternatively, change points can be identified using threshold regression (figure 1*d*) or by finding the locations of knots (which separate sections to each different polynomials are fitted) in GAM models [66].

## 4. From inferring to forecasting

To forecast future change, it is crucial to understand how biodiversity changes through time. Having taken into account how measurement error affects perception of biodiversity change, knowledge of the patterns of correlation (autocorrelation and cross-correlation with predictor variables) can be used for this purpose. Incorporating autocorrelation is a parsimonious approach to improving the precision of forecasts by including the effects of unmeasured factors, which are reflected in autocorrelation patterns.

Forecasting can be accomplished in three main ways. First, temporal trends can be extrapolated into the future. The slope of a line fitted to the time series (using GLS, GAM or ARIMA models, for example) is indicative of the trend in the time series. However, an understanding of the patterns of temporal autocorrelation is crucial to gauge how uncertainty scales with time lags, and hence how far into the future it is reasonable to extend predictions. An example of a forecasted trend are extinctions caused by habitat loss as estimated from the species–area relationship [69], which are predicted to occur over an extended period of time, with an extinction debt persisting well into the future [70]. This extinction debt over time can be forecasted and intervention windows for conservation action to prevent extinction estimated [71].

Second, biodiversity can be modelled as a function of covariates, which we may be able to predict more accurately than biodiversity itself, and hence obtain indirect predictions of future biodiversity. Again, regression models such as GLS or GAMs can be particularly useful in this endeavour, but it is important to consider how temporal autocorrelation can cloud our understanding of cross-correlations. An example of predictions based on forecasted covariates is the prediction that climate change may cause the extinction of many endemic species in Australian tropical rainforests [72].

Third, process-based ecosystem models can be used to project future abundance and distribution of biodiversity [73]. Incorporating the time axis and understanding the effects of time lags could extend our ability to model biodiversity as a function of covariates and thus to predict, but is not yet used in, species distribution models (W. Thuiller 2011, personal communication). The accuracy of these approaches depends heavily on how completely we understand the mechanics of the community, and tends to decrease with increasing complexity. A recent comparison of predictions by different models at a global-scale highlights the level of uncertainty in these forecasts and the extent to which incomplete ecological knowledge contributes to this uncertainty [74]. For example, predicted extinction rates vary nearly twofold, depending on poorly understood migration rates [75]. Ultimately, these models can only be as good as our empirical understanding of the ecological mechanisms involved in biodiversity change. To predict long-term, large-scale change, we need biodiversity time series at comparable scales.

The most serious difficulty with forecasting biodiversity change is that many past changes have been neither gradual nor linear. Examples of drastic changes that fundamentally altered biodiversity and ecosystem function include the mass extinctions evident from the fossil record, with a mass extinction event possibly currently underway [76], and ecosystem phase-shifts between alternative stable states, such as coral dominated and algae dominated reefs [77]. Additionally, ecosystems often show path dependence (hysteresis), in which restoring conditions before the tipping point is not sufficient to reverse a phase-shift [78]. An urgent area of research, in which temporal patterns of biodiversity are crucial, is learning to recognize early warning signs of drastic changes in ecosystems before they occur. Specifically, analysing frequency patterns in autocorrelation may provide important clues (J. Ardron 2011, personal communication). Fluctuations in ecological communities have long been recognized as containing important information regarding ecosystem stability [79]. Ecosystems tend to recover more slowly from perturbations and show increased variance in temporal patterns before undergoing a phase-shift to an alternate basin of attraction [6]. Specifically, studying autocorrelation patterns in small grain time series that include phase-shifts will provide deeper insights into these patterns. Examining the generality of changes in autocorrelation patterns prior to phase-shifts may provide important tools to anticipate drastic biodiversity change, much like monitoring seismic activity helps predict major earthquakes.

Most statistical methods that are used to forecast future trajectories of biodiversity implicitly assume that the mechanisms driving historical and recent trends continue into the future, albeit with new levels for some covariates. However, drastic changes often involve pressures upon ecosystems, generating novel systems [80] that function differently, as the pool of functional traits changes [81], and combinations of environmental variables arise that have no contemporary analogues [82]. In many cases, palaeo-climates encompass a greater range of projected conditions and may provide important clues for expected biotic responses [83]. These issues create challenges for predicting biodiversity trends that require us to understand the mechanisms driving diversity and call for placing greater statistical weight on datasets that better represent the anticipated change.

## 5. Conclusions

Availability of long-term, large-scale, high-resolution data is the single most important factor limiting progress in understanding temporal patterns in biodiversity. Given the difficulty of obtaining data from the past, we reiterate the appeal to preserve data and associated metadata in publicly accessible archives [84]. Public databases of biodiversity records are providing unprecedented insight into large-scale, long-term patterns (e.g. Paleobiology database—http://paleodb.org, Global Biodiversity Information Facility—http://data.gbif.org/). Establishing standards for meta-information should ensure that future scientists can not only access but also take full advantage of the data we are now collecting [85]. The challenges that arise from dealing with historical data (see the electronic supplementary material) should help signal pitfalls to avoid when making contemporary data available.

If possible, data should be collected using methods, grain and sampling effort that allow linking to other data sources, such as palaeo and historical data. Achieving standardization of methods will facilitate integration of multiple sources of contemporary data. Although ecologists should always strive to collect data as accurately as possible, incomplete or partial data can be better than no data at all. Imperfect data at relevant spatial and temporal scales (e.g. range maps from floras) allow answering questions unapproachable with high precision data at short timescales or with time series that take highly degraded states as the baseline [18]. Exploring non-traditional sources of data, such as archaeological deposits, historical images and traditional knowledge passed orally through generations [86], and collating different sources of data may help address previously intractable questions.

The unique features of temporal data should be recognized, accepted and used as advantages rather than treated as nuisances. These include the general lack of boundaries, uni-dimensionality, inherent autocorrelation and directionality. Rather than coercing temporal data into restrictive assumptions for analysis, methods that treat these characteristics as part of the pattern should be considered. The study of autocorrelation and frequency analysis of time series and their relationship with ecosystem stability are areas that we believe will prove fruitful. Measurement error should be minimized and, when possible, estimated, particularly given the potential additional sources of error in temporal data. We should consider the spatial context of time series, the temporal context of spatial data, and types and rates of change expected in fully spatio-temporal contexts.

Finally, forecasts of biodiversity change should recognize that the future is never a strict repetition of the past but appreciate that the past sheds light on how life on earth has dealt with immense challenges and how biodiversity responds to critical transitions.
